# Community case management of malaria in Western Kenya: performance of community health volunteers in active malaria case surveillance

**DOI:** 10.1186/s12936-023-04523-4

**Published:** 2023-03-08

**Authors:** Wilfred Ouma Otambo, Kevin O. Ochwedo, Collince J. Omondi, Ming-Chieh Lee, Chloe Wang, Harrysone Atieli, Andew K. Githeko, Guofa Zhou, James Kazura, John Githure, Guiyun Yan

**Affiliations:** 1 International Centre of Excellence for Malaria Research, Tom Mboya University, University of California Irvine Joint Lab, Homa Bay, Kenya; 2 Program in Public Health, University of California Irvine, Irvine, CA USA; 3 Centre for Global Health Research, Kenya Medical Research Institute, Kisumu, Kenya; 4 Department of Pathology, School of Medicine, Case Western Reserve University, Cleveland, OH USA

**Keywords:** Malaria, Community health volunteers, Community, Case management, Kenya

## Abstract

**Background:**

In western Kenya, not all malaria cases are reported as stipulated in the community case management of malaria (CCMm) strategy. This underreporting affects the equity distribution of malaria commodities and the evaluation of interventions. The current study aimed to evaluate the effectiveness of community health volunteers’ active case detection and management of malaria in western Kenya.

**Methods:**

Cross-sectional active case detection (ACD) of malaria survey was carried out between May and August 2021 in three eco-epidemiologically distinct zones in Kisumu, western Kenya: Kano Plains, Lowland lakeshore and Highland Plateau. The CHVs conducted biweekly ACD of malaria household visits to interview and examine residents for febrile illness. The Community Health Volunteers (CHVs) performance during the ACD of malaria was observed and interviews done using structured questionnaires.

**Results:**

Of the total 28,800 surveyed, 2597 (9%) had fever and associated malaria symptoms. Eco-epidemiological zones, gender, age group, axillary body temperature, bed net use, travel history, and survey month all had a significant association with malaria febrile illness (p < 0.05). The qualification of the CHV had a significant influence on the quality of their service. The number of health trainings received by the CHVs was significantly related to the correctness of using job aid (χ^2^ = 6.261, df = 1, *p* = 0.012) and safety procedures during the ACD activity (χ^2^ = 4.114, df = 1, *p* = 0.043). Male CHVs were more likely than female CHVs to correctly refer RDT-negative febrile residents to a health facility for further treatment (OR = 3.94, 95% CI = 1.85–5.44, *p* < 0.0001). Most of RDT-negative febrile residents who were correctly referred to the health facility came from the clusters with a CHV having 10 years of experience or more (OR = 1.29, 95% CI = 1.05–1.57, *p* = 0.016). Febrile residents in clusters managed by CHVs with more than 10 years of experience (OR = 1.82, 95% CI = 1.43–2.31, *p* < 0.0001), who had a secondary education (OR = 1.53, 95% CI = 1.27–1.85, p < 0.0001), and were over the age of 50 (OR = 1.44, 95% CI = 1.18–1.76, *p* < 0.0001), were more likely to seek malaria treatment in public hospitals. All RDT positive febrile residents were given anti-malarial by the CHVs, and RDT negatives were referred to the nearest health facility for further treatment.

**Conclusions:**

The CHV’s years of experience, education level, and age had a significant influence on their service quality. Understanding the qualifications of CHVs can assist healthcare systems and policymakers in designing effective interventions that assist CHVs in providing high-quality services to their communities.

**Supplementary Information:**

The online version contains supplementary material available at 10.1186/s12936-023-04523-4.

## Background

Kenya is currently ramping up malaria control efforts in order to reduce the disease's burden and eventually eliminate malaria. Despite increased efforts by the Ministry of Health to scale up intervention strategies, the malaria burden in Kenya remains high [1–3]. Accurate, reliable and early diagnosis followed by effective malaria treatment is the key to reducing malaria burden [4, 5]. The ultimate goal of the Kenya National Malaria Control Programme is to provide access to effective malaria preventive interventions while also drastically lowering the incidence and mortality of malaria among those who live in malaria-risk areas [3]. The burden of malaria is exacerbated by challenges in accessing healthcare facilities, especially in rural areas, where there is often limited access to health services.

Access to effective malaria treatment and prevention has been hampered by accessibility, availability, and affordability of diagnostic and treatment services [6]. Topographic features of the local landscape major correlates with malaria infection in the Lake Victoria area of western Kenya [7]. Clinical malaria incidence remains highest in the Lakeshore of western Kenya despite high long-lasting insecticidal nets (LLINs) coverage [4]. Fever is the most common symptom of clinical malaria, and its severity drives people to seek treatment at health care facilities [8]. Only a small percentage of the residents with fever seek treatment at a health facility, with the vast majority self-medicating at home [9]. The fact that the majority of febrile residents seeking malaria treatment use over-the-counter medication without a confirmed laboratory test and prescription is a major concern, as anti-malarial overuse may promote drug resistance to current anti-malarial drugs [4]. Treatment that is ineffective or fails to treat true blood stage infections can result in increased healthcare costs [5, 10]. As a result, the emphasis should be on the accuracy of community malaria diagnosis and treatment in order to reduce the persistence of malaria febrile illness in the community. Emphasis should be active case surveillance of malaria.to optimize the accuracy of malaria diagnosis and treatment at the community.

Active malaria case surveillance strategy is essential for effective malaria control in areas where the disease is endemic [11]. Active surveillance aids in the identification of potential malaria risk factors, and by analysing the data, public health experts can identify any environmental factors that may be contributing to the persistence of malaria [7]. The effectiveness of active surveillance is determined by the system's implementation as well as how well it is monitored and maintained. If the system is not effectively monitored and maintained, it may be unable to detect any increase in malaria cases or potential risk factors [12]. In Western Kenya, community health volunteers (CHVs) have been engaged in active malaria case surveillance through routine visits to households to identify and report suspected malaria cases [4]. This approach ensures that malaria cases are detected early, and appropriate treatment can be provided promptly. Active surveillance of malaria necessitates the collection of reliable and accurate data in order to track disease trends, inform public health policies, and provide an evidence base for healthcare providers to make treatment and community case management of malaria decisions.

Community case management of malaria (CCMm) strategy aims to improve access to and quality of malaria treatment while also reaching a larger proportion of the population, particularly the poorer segments of society, with primary health care [13]. Bringing care into the community may remove barriers to seeking care in health facilities, such as distance, transportation costs, travel time, and fixed operating hours. The approach involves training community health workers, such as CHVs, to diagnose and treat uncomplicated malaria cases in their communities [14]. As CHVs should be more acceptable sources of care for villagers than facility-based personnel, well-trained and supervised CHVs can provide prompt and adequate treatment and care to patients close to their homes [15]. In order to educate and motivate their community members to seek early malaria treatment, CHVs should have a basic understanding of malaria, its transmission, and its signs and symptoms, as well as good communication skills. However, in many areas, the use of CHV services remains suboptimal, with families unaware that they exist and inconsistency in drug supplies [16]. The services of the CHVs are critical to the success of CCMm, and any gaps in their needs must be identified for optimal performance and subsequent malaria burden reduction.

Despite the proven effectiveness of CCM and active malaria case surveillance, there are still challenges in scaling-up and sustaining their implementation in many parts of Western Kenya due to insufficient resources and lack of support from the community and health system, as well as a lack of the necessary knowledge and skills to provide high-quality care [16]. Identifying and addressing these issues is critical for the successful implementation and scaling up of CCM and active malaria case surveillance in Western Kenya. The purpose of this study is to assess CHVs' active case detection and management of malaria in western Kenya, as well as gaps in strengthening CCMm.

## Methods

### Study area and design

The study was carried out in the Nyakach and Muhoroni Sub-County of Kisumu County in western Kenya near the shores of Lake Victoria at latitude 0.333333°S and longitude 34.99100°E**.** Based on malaria prevalence and incidence, malaria vector densities and topographical features [4, 7, 17, 18], the study area was divided into three eco-epidemiological zones: Kano Plains, Lowland Lakeshore and Highland Plateau. The Kano Plains is characterized by a shallow inland plain with an elevation of about 1150 m to 1200 m, frequented by flooding during the rainy season, with rice irrigation and sugarcane plantation as the main cash crops. The Lowland Lakeshore and Highland Plateau eco-epidemiological zones have previously been described [7]. Each ecological zone was further randomly selected with 24 clusters for study. Based on the administrative village or natural boundary, such as a river or major road, a cluster was delineated with approximately 1 km2 area. Each study area had around 150 households, with an average of about 400 residents under the management of a CHV. Malaria prevalence in the study area is estimated to be around 18% [7], with the common vectors of malaria transmission being *Anopheles funestus* and *Anopheles gambiae* [18]*.*

### Study participation and data collection

#### Active case detection of malaria

Cross-sectional community surveys were carried out between May to August 2021 when malaria transmission was at its peak in Western Kenya [4, 7, 18]. The CHVs were trained on recording febrile cases in each household, taking blood samples for RDT, and preparing dry blood spots for real-time-PCR (RT-PCR) analysis. A febrile malaria case was defined as an individual with fever (axillary temperature ≥ 37.5 °C) at the time of examination or complaints of fever and other nonspecific symptoms 1–2 days prior to examination [19]. The CHVs used an active case detection (ACD) questionnaire to interview residents about their fever status. Febrile residents’ age, sex, and active fever, fever days, treatment-seeking behaviours, primary occupation, travel history, and bed net usage, health insurance coverage, transportation method to the health facility, and reasons for delaying in treatment were collected in the questionnaire. The questionnaire results were reviewed daily by team supervisors for quality assurance.

Finger-prick blood samples were taken from febrile cases for parasite examination with ultra-sensitive Alere^®^ malaria RDT (Reference number: 05FK140, Republic of Korea) and RT-PCR on dry blood spots. The samples were then transported to the International Centre of Excellence for Malaria Research (ICEMR) at the University of California Irvine-Tom Mboya University Joint Laboratory in Homa Bay, Kenya [4, 5], for further analysis. The Chelex resin (Chelex-100) saponin method was used with minor modifications [20]. Primers and probes specific to *Plasmodium* species were used to target 18S ribosomal RNA [21] to confirm the presence of parasite DNA on QuantStudio™ 3 Real-Time PCR.

#### Assessment of CHVs qualification on quality of service

A total of 72 CHVs working with the Ministry of Health Kenya in Kisumu County in the Kano plains, Lakeshore zone and the Highland Plateau zones were surveyed and sought to ascertain their performance of CCMm. Questionnaire was used by the project team to collected information on CHVs age, gender, income-generating activity, years of experience, education level, and the community health and professional trainings attended. A service quality questionnaire was used by the project team to interview all 72 CHVs, and another observational checklist was used to evaluate the preparedness and how they performed the malaria diagnosis and treatment during the ACD survey. The assessment of the CHVs quality of service and CCMm were standardized based previous studies [22–24]. The service quality was defined as the correctness of using ACD job aid, classification of malaria symptoms, experience with commodity stock-out, and safety procedure to perform the ACD visits. These criteria were evaluated using a checklist as satisfactory or unsatisfactory.

The CHV assessment of malaria diagnosis included the following aspects: maintaining a good rapport with residents and community acceptance, the correctness of taking body temperature, recording ACD report, explaining the necessity of malaria testing, adequate testing preparation, labelling test kit, using the glove, disinfection for pricking, collecting blood samples, reading results at the appropriate time, interpreting results, and communicating results to the patients. The evaluation of malaria treatment and management included these aspects: following the MOH treatment guidelines to administer the AL in the appropriate dosage, explaining treatment duration, following up on the treatment of febrile residents, proper waste disposal, difficulties of referral, and the need for supportive supervision. An additional 12 CHVs from the study clusters were pretested with those questionnaires to ensure the completeness of survey processes and data quality.

### Data analysis

Data were analysed using IBM SPSS Statistics (version 21). The demographic profiles of the study participants were described using descriptive statistics. The multivariate binary logistic regression model was used for risk factor analysis. Chi square test and Odds ratio, determined the association between the CHVs qualification and the quality of service. Regression analysis determined the influence of CHVs' qualifications on treatment seeking patterns of febrile residents. For all analyses, *p* ≤ 0.05 was considered statistically significant. Additional file 1: Table S1 categorizes the demographic characteristics of CHVs and febrile residents.

## Results

### Febrile resident’s demographic information

A total of 2597 (9%) residents with fever and associated malaria symptoms from 10,800 households with a total population of 28,800 agreed to participate in the study. The demographic information of the febrile residents is summarized in Table 1.

**Table 1:** Descriptive statistics of the febrile residents’ demographic information

Parameter	Options	Enrollment (%)		Eco-epidemiological Zone (%)			*p*-value
				Kano Plains	Lowland Lakeshore	Highland Plateau	
Enrolled resident		2597		954	807	836	
Sex	Male	1104 (42.5)		414 (43.4)	355 (44.0)	335 (40.1)	0.216
	Female	1493 (57.5)		540 (56.6)	452 (56.0)	501 (59.9)	
Age	< 5 years old	417 (16.1)		155 (16.2)	130 (16.1)	132 (15.8)	0.065
	5 ~ 14 years old	974 (37.5)		390 (40.9)	289 (35.8)	295 (35.3)	
	≥ 15 years old	1206 (46.4)		409 (42.9)	388 (48.1)	409 (48.9)	
Education level	Never attended school	225 (8.7)		48 (5.0)	59 (7.3)	118 (14.1)	< 0.0001
	Pre-school age	350 (13.5)		122 (12.8)	106 (13.1)	122 (14.6)	
	Primary	1574 (60.6)		622 (65.2)	498 (61.7)	454 (54.3)	
	Secondary	332 (12.8)		124 (13.0)	106 (13.1)	102 (12.2)	
	College & above	116 (4.5)		38 (4.0)	38 (4.7)	40 (4.8)	
Community health training & workshops attended	Farmer	535 (20.6)		218 (22.9)	189 (23.4)	128 (15.3)	< 0.0001
	Small scale business	234 (9.0)		91 (9.5)	59 (7.3)	84 (10.0)	
	Unemployed	108 (4.2)		17 (1.8)	32 (4.0)	59 (7.1)	
	Student	1447 (55.7)		584 (61.2)	435 (53.9)	428 (51.2)	
	Non-school child	110 (4.2)		25 (2.6)	37 (4.6)	48 (5.7)	
	Others	163 (6.3)		19 (2.6)	55 (6.8)	89 (10.6)	

### Risk factors associated with malaria febrile illness

**Fig. 1:**
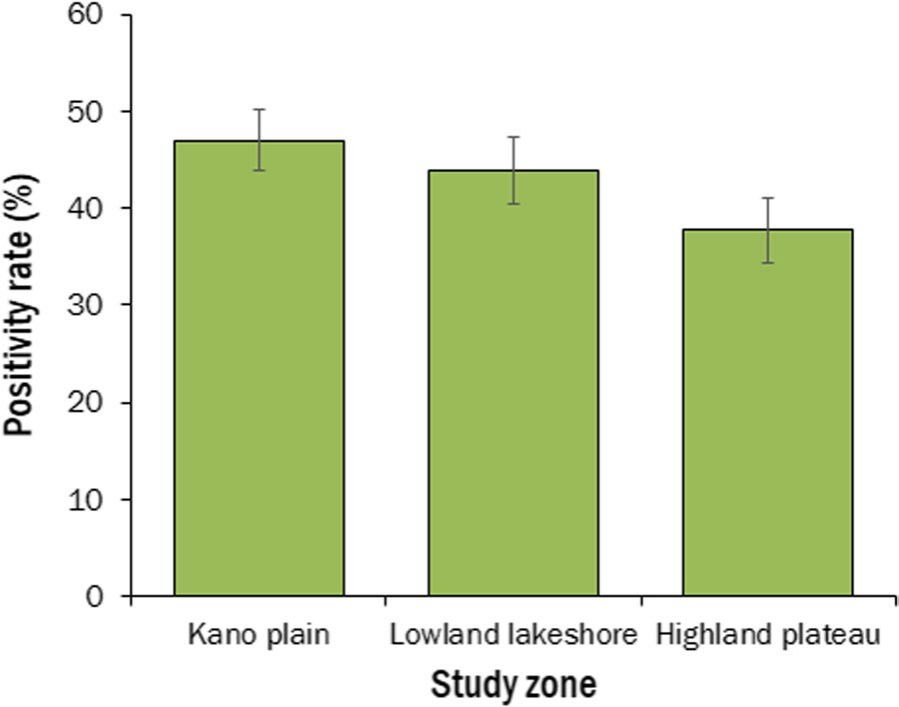
RDT positivity rates across eco-epidemiological zones. Error bar represents 95% confidence interval

Malaria febrile illness differed significantly across eco-epidemiological zones (χ^2^ = 16.006, df = 2, *p* < 0.0001). The RDT positivity rate was highest in the Kano Plains at 47.0% (448/954), followed by the Lowland Lakeshore at 43.9% (354/807) and the Highland Plateau at 37.7% (315/836) (Fig. 1).

The zone of residence, gender, age group, axillary body temperature, bed net usage, travel history, and survey month were significantly association with malaria febrile illness (Table 2).

**Table 2:** Risk factor associated with malaria febrile illness

Risk factors	Category	Adjusted odd ratio	95% CI	*p*-value
Zones	Kano Plains	1.41	1.17–1.71	< 0.0001
	Lowland Lakeshore	1.28	1.05–1.56	0.016
	Highland Plateau	Ref.		
Sex	Female	0.79	0.67–0.92	0.004
	Male	Ref.		
Age group	< 5 years old	1.87	1.49–2.35	< 0.0001
	5–< 15 years old	2.22	1.87–2.66	< 0.0001
	≥ 15 years old	Ref.		
Temperature	< 37.5 °C	0.52	0.44–0.62	< 0.0001
	≥ 37.5 °C	Ref.		
Bednet usage	No net	1.60	1.13–2.27	0.008
	Use net	Ref.		
Travel history	No	0.62	0.42–0.92	0.017
	Yes	Ref.		
Occupation/ income generating activity	Farmer	0.96	0.65–1.42	0.844
	Commercial sales	0.71	0.45–1.12	0.713
	Unemployed	1.47	0.88–2.46	0.143
	Child younger than working age	1.79	1.13–2.84	0.012
	Others	Ref.		
Month	May	0.77	0.60–0.98	0.035
	June	0.79	0.62–0.99	0.039
	July	1.41	1.13–1.75	0.002
	August	Ref.		

### Demographics information of the CHVs

A total of 72 CHVs were recruited for the study. The majority of CHVs in the study zones were females 65 (90%), over the age of 52 (72%), had more than 10 years of experience as a CHVs 43 (60%), had a secondary education 37 (51%), and had received more than ten trainings 44 (61%) on community health work (Table 3).

**Table 3:** Demographic information of CHVs

Parameter	Options	Enrollment n (%)		Eco-epidemiological Zone		
				Kano Plains n (%)	Lowland Lakeshore n (%)	Highland Plateau n (%)
Enrolled CHV		72		24	24	24
Sex	Male	7 (10)		2 (8)	2 (8)	3 (12)
	Female	65 (90)		22 (92)	22 (92)	21 (88)
Age	< 50 years old	20 (28)		6 (25)	3 (13)	11 (46)
	≥ 50 years old	52 (72)		18 (75)	21 (88)	13 (54)
Years of experience as a CHV	< 10 years	29 (40)		7 (29)	4 (17)	10 (42)
	≥ 10 years	43 (60)		17 (71)	20 (83)	14 (58)
Education Level	Primary	35 (49)		13 (54)	10 (42)	12 (50)
	Secondary	37 (51)		11 (46)	14 (58)	12 (50)
Income generating activity	Farmer	56 (78)		16 (67)	20 (23)	20 (83)
	Small scale business	12 (17)		6 (25)	2 (8)	4 (17)
	Others	4 (6)		2 (8)	2 (8)	0 (0)
Community health training & workshops attended	< 10 Training	28 (39)		9 (38)	7 (29)	12 (50)
	≥ 10 Training	44 (61)		15 (63)	17 (71)	12 (50)

### Influence of CHVs qualifications on the quality of service

The CHV years of experience, education level, health training received, age and gender significantly influenced their quality of service. The number of community health trainings received by the CHVs was significantly related to the correctness of using job aid (χ^2^ = 6.261, df = 1, *p* = 0.012) and safety procedures during the ACD activity (χ^2^ = 4.114, df = 1, *p* = 0.043). Regardless of age, gender, experience, education, or received training, all 72 CHVs correctly classified malaria symptoms (Additional file 1: Table S2).

Male CHVs were more likely than female CHVs to correctly refer RDT-negative febrile residents to a health facility for further treatment (OR = 3.94, 95% CI = 1.85–5.44, *p* < 0.0001). Most of RDT-negative febrile residents who were correctly referred to the health facility came from the clusters with a CHV having 10 years of experience or more (OR = 1.29, 95% CI = 1.05–1.57, *p* = 0.016). Conversely, most of RDT-positive febrile residents who had received anti-malarial medication came from the clusters with a CHV having more than ten years of experience (OR = 2.49, 95% CI = 1.90–3.27, *p* < 0.0001) and had secondary education (OR = 1.95, 95% CI = 1.60–2.37, *p* < 0.0001) (Table 4).

**Table 4:** Association of CHV’s correct referral of RDT negatives and antimalarial treatment of positives

Parameter	Options	Correct referral of RDT-negatives			Correct treatment of RDT-positives		
		Odd ratio	95% CI	*p*-value	Odd ratio	95% CI	*p*-value
Years of experience	> 10 years old	1.29	1.05–1.57	0.016	2.49	1.90–3.27	< 0.0001
	< 10 years old	Ref.			Ref.		
Education level	Secondary	0.85	0.72–1.01	0.068	1.95	1.60–2.37	< 0.0001
	Primary	Ref.			Ref.		
Training Received	> 10	0.40	0.33–0.48	< 0.0001	0.64	0.51–0.80	< 0.0001
	< 10	Ref.			Ref.		
Sex	Male	3.94	1.85–5.44	< 0.001	0.54	0.33–0.87	0.012
	Females	Ref.			Ref.		
Age	> 50 years	0.99	0.83–1.17	0.887	1.21	0.98–1.48	0.073
	< 50 years	Ref.			Ref.		

*RDT* Rapid Diagnostic Test, *CHV* Community Health Volunteer, *Ref.* Refrence category

### Influence of CHVs qualifications on malaria febrile residents’ treatment-seeking pattern

The CHVs' qualifications significantly influenced treatment-seeking patterns of febrile residents in their clusters. Febrile residents in clusters managed by CHVs with more than 10 years of experience were more likely to seek treatment at health facility (OR = 1.82, 95% CI = 1.43–2.31, *p* < 0.0001) but less likely to do nothing (OR = 0.56, 95% CI = 0.46–0.68, *p* < 0.0001) compared to those in clusters managed by CHVs with less than 10 years. Similarly, in clusters where the CHV had secondary education, febrile residents were more likely to visit health facilities (OR = 1.53, 95% CI = 1.27–1.85, p < 0.0001), but less likely to do nothing (OR = 0.78, 95% CI = 0.66–0.92, *p* = 0.003) compared to clusters where the CHV had primary education. In clusters with male CHVs, febrile residents were more likely to do nothing (OR = 2.33, 95% CI = 1.69–3.21, *p* < 0.0001) and less likely buy drugs from the drug shops (OR = 0.28, 95% CI = 0.17–0.47, *p* < 0.0001) compared to clusters with female CHVs. In clusters with a CHV aged 50 or above, febrile residents were more likely to seek treatment at the health facility (OR = 1.44, 95% CI = 1.18–1.76, *p* < 0.0001), but less likely to buy drugs from the drugs shops (OR = 0.66, 95% CI = 0.55–0.79, *p* < 0.0001) compared to those clusters with CHVs in age below 50 years old (Table 5).

**Table 5:** Influence of CHVs qualifications on malaria febrile resident’s treatment seeking patterns

Parameter	Options	Residents’ treatment patterns							
		Hospital		Drug shop		Traditional medication		Do nothing	
		Odd ratio (95% CI)	*p*-value	Odd ratio (95% CI)	*p*-value	Odd ratio (95% CI)	*p*-value	Odd ratio (95% CI)	*p*-value
Years of experience	≥ 10 years	1.82 (1.43–2.31)	< 0.0001	1.35 (1.08–1.70)	0.009	0.71 (0.51–0.99)	0.044	0.56 (0.46–0.68)	< 0.0001
	< 10 years	Ref.		Ref.		Ref.		Ref.	
Education level	Secondary	1.53 (1.27–1.85)	< 0.0001	0.86 (0.72–1.03)	0.102	1.18 (0.88–1.58)	0.276	0.78 (0.66–0.92)	0.003
	Primary	Ref.		Ref.		Ref.		Ref.	
Training Received	≥ 10	0.53 (0.43–0.66)	< 0.0001	0.89 (0.73–1.09)	0.258	0.60 (0.42–0.88)	0.008	1.98 (1.65–2.37)	< 0.0001
	< 10	Ref.		Ref.		Ref.		Ref.	
Sex	Male	1.44 (1.01–2.06)	0.044	0.28 (0.17–0.47)	< 0.0001	0	0.995	2.33 (1.69–3.21)	< 0.0001
	Females	Ref.		Ref.		Ref.		Ref.	
Age	≥ 50 years old	1.44 (1.18–1.76)	< 0.0001	0.66 (0.55–0.79)	< 0.0001	1.37 (1.00–1.87)	0.050	1.00 (0.85–1.18)	0.995
	< 50 years old	Ref.		Ref.		Ref.		Ref.	

### Factor associated with febrile residents’ decision to seek malaria treatment

The CHVs evaluated 754 of the total 2597 febrile residents to assess determinants of the decision to seek treatment. The decision to seek treatment was significantly associated with the reasons for the delay in treatment (χ^2^ = 67.633, df = 4, *p* < 0.0001), transportation method to the health facility (χ^2^ = 75.316, df = 6, *p* < 0.0001), and availability of medical insurance coverage (χ^2^ = 24.125, df = 2, *p* < 0.0001). Compared to the Lowland Lakeshore and Highland Plateau, the affordability of treatment (76.4%, 201/263) and the severity of disease (11.8%, 31/263) in the Kano Plain contributed to the delay in seeking treatment. In addition, Kano Plain residents (60.5%, 159/263) preferred walking, while Highland Plateau residents (53.4%, 125/234) preferred motorbikes and Lowland Lakeshore residents (15.6%, 40/257) preferred vehicles in the transportation method question. In comparison to the Kano Plain (8.7%, 23/263) and the Highland Plateau (12.0%, 28/234), the Lowland Lakeshore (23.3%, 60/257) had the highest insurance coverage (Table 6).

**Table 6:** Determinants of decision to seek treatment among malaria febrile residents

Parameter	Options	Eco-epidemiological zones						χ^2^ value	*p*-value
		Kano plains		Lowland lakeshore		Highland plateau			
Study subjects		263		257		234			
Time taken before treatment (%)	1 day	127	(48.3)	146	(56.8)	123	(52.6)	5.498	0.240
	2 days	97	(36.9)	83	(32.3)	74	(31.6)		
	> 2 days	39	(14.8)	28	(10.9)	37	(15.8)		
Reason for delay in treatment (%)	Affordability	201	(76.4)	146	(56.8)	128	(54.7)	67.633	< 0.0001
	Distance	24	(9.1)	72	(28.0)	55	(23.5)		
	Severity of disease	31	(11.8)	18	(7.0)	14	(6.0)		
	Others	7	(2.7)	21	(8.2)	37	(15.8)		
Mode of transport to health facility (%)	Walk	159	(60.5)	94	(36.6)	102	(43.6)	75.316	< 0.0001
	Motorbike	103	(39.2)	123	(47.9)	125	(53.4)		
	Vehicle	1	(0.4)	40	(15.6)	7	(3.0)		
Medical insurance cover (%)	Yes	23	(8.7)	60	(23.3)	28	(12.0)	24.125	< 0.0001
	No	240	(91.3)	197	(76.7)	206	(88.0)		

## Discussion

The current study evaluated the effectiveness of CHVs in active malaria surveillance and CCMm in rural community of western Kenya. In the current study ACD survey conducted by the CHVs, eco-epidemiological zones, gender, age group, axillary body temperature, bednet use, travel history, and survey month were significantly association with malaria febrile illness. The CHV’s years of experience, education level, and age had a significant influence on their service quality. The CHVs correctly classified malaria symptoms, used the ACD malaria job aid satisfactorily, promptly reported commodity stock-outs, and followed safety precautions during the ACD. The number of health trainings received by the CHVs was significantly related to the correctness of using job aid and safety procedures during the ACD activity. Male CHVs were more likely than female CHVs to correctly refer RDT-negative febrile residents to a health facility for further treatment. Most of RDT-negative febrile residents who were correctly referred to the health facility came from the clusters with a CHV having 10 years of experience or more. Febrile residents in clusters managed by CHVs with more than 10 years of experience, secondary education, and were over the age of 50, were more likely to seek malaria treatment in public hospitals. All RDT positive febrile residents were given anti-malarial by the CHVs and RDT negatives were referred to the nearest health facility for further treatment.

In the current study, CHVs correctly handled malaria febrile illness, using RDT for malaria, and uncomplicated malaria prescriptions well. This included the understanding of malaria as well as community awareness of disease control and prevention. The CHVs administered AL to all febrile residents who tested positive for malaria by RDT. Residents who tested negative for RDT were referred to the health facility for further treatment. Similar to the current study in western Kenya, the evaluation of the effectiveness of CHV active case detection and management of malaria found that CHVs detected a high proportion of malaria cases by being able to accurately identify and treat malaria cases using RDTs and appropriately treated them with artemether-lumefantrine as well as effectively refer severe cases to higher-level facilities [25, 26]. Furthermore, the use of CHVs in malaria control and management resulted in a significant reduction in malaria prevalence [24].

The goal of community malaria case management is to reach a larger proportion of the population, particularly the poorer segments of society, with primary health care [13]. Despite the fact that approximately 80% of missed malaria cases in the community do not seek treatment at a health facility, bringing care into the community may remove barriers to seeking care in health facilities, such as distance, transportation costs, travel time, and fixed operating hours. With the majority of febrile residents not seeking treatment at a health facility, as reported in this and another study [4], infrastructure support for CHVs will result in a reduction in anti-malarial misuse without a confirmed laboratory test and the missed out underreported malaria cases.

CHVs with more years of experience in diagnosing and treating malaria may have a higher level of expertise and knowledge, which resulted in higher quality of service as observed in the current study. The ability of CHVs to build and maintain trust with the communities they serve is critical to their success in providing healthcare services. More experienced CHVs may have a better understanding of the community's needs, preferences, and cultural beliefs. As a result, they may be more effective in providing community members with relevant health promotion information, advice, and support on malaria prevention and treatment activities [24]. Their knowledge can also assist them in identifying high-risk groups and tailoring interventions to their specific needs. This level of tailored intervention can help the CHV and the community build trust. According to a study conducted in western Kenya, CHVs with more years of experience had better knowledge of malaria prevention and treatment, were more trusted by the community, and had better communication skills, which resulted in increased community participation in malaria prevention and treatment activities [27]. A study in western Kenya showed that CHVs with more years of experience in malaria diagnosis and treatment were more accurate in diagnosing and treating malaria than those with less experience [28]. Experienced CHVs were more likely to use RDTs and adhere to treatment guidelines [29]. The current study also found that the more experienced the CHV, the more likely the febrile residents were to be referred to a health facility for further medical attention, and the less experienced the CHV, the more likely the febrile cases in their clusters were to use traditional medication and do nothing when they had a fever.

The current study discovered that CHVs in the study area performed proper malaria diagnostics, treated febrile residents, and followed RDT interpretation of results, and that this was related to the CHV's education. CHVs with higher levels of education may have better understanding of health concepts and be able to communicate more effectively with the community. This can lead to improved health outcomes, greater satisfaction with their services, more accurate diagnosis and treatment of malaria, as well as better management of any associated symptoms. The CHVs with at least a secondary education are more likely to provide appropriate treatment for malaria and refer severe cases to formal healthcare facilities [30].

CHVs who receive regular and comprehensive training on health topics, such as disease prevention and health promotion, may be more effective in their role. Comprehensive training on malaria diagnosis and treatment, how to use RDTs and how to properly administer anti-malarial drugs may be more effective in providing accurate and appropriate treatment to febrile patients by the CHVs. Better trainings will equip the CHV with confident in their ability to provide quality services in the CCMm. CHVs who had received adequate malaria diagnosis and treatment training were more likely to diagnose and treat malaria correctly, and those who received regular supportive supervision were more likely to adhere to treatment guidelines [31].

CHVs of different ages and genders may have varying levels of community trust and credibility, which can affect their ability to effectively diagnose and treat malaria. Older CHVs may be perceived as having more life experience and wisdom, which can contribute to their community credibility and trustworthiness. Older people are treated with more respect and are thought to be more reliable and trustworthy than younger people. These older CHVs have improved communication skills, more knowledgeable about the community’s social and economic context, and have built strong trusting relationships with community members [32]. As a result, older CHVs may be more effective at building community trust, which may lead to increased adherence to malaria prevention and treatment recommendations. Furthermore, older the CHV, the more experience they have and thus febrile residents could take malaria information and knowledge from them seriously.

The majority of CHVs in the study site were females, 90% of which is consistent with the general trend in western Kenya [33]. Male CHVs may be more effective at engaging men in health promotion activities, while female CHVs may be more effective at engaging women and children. In contrast to the current study, a study done in Nigeria found that female CHVs were more likely than male CHVs to correctly diagnose and treat malaria, and younger CHVs were more likely to follow treatment guidelines than older ones [34].

As demonstrated in the current study, CHVs' qualifications have a significant impact on the quality of service and treatment seeking patterns of febrile residents within their clusters. Working with CHVs lowers the cost of accessing malaria diagnosis and treatment services, as evidenced by CHVs competently offering malaria diagnosis, treating uncomplicated cases, and referring complicated cases to nearby health facilities for further management. Based on the CHVs' optimal performance in classifying malaria symptoms, promptly reporting commodity stock-outs, and good ACD performance, it is possible that the CHVs could be used to address gaps in the persistence of malaria cases in both endemic and non-endemic areas with supportive supervision, more trainings, and improved supply of testing kits and drugs. Therefore, when managing malaria cases in the community, the proper training, years of experience, education level, and age of the CHVs should all be taken into account for optimal performance of CCMm.

## Conclusion

The CHV years of experience, education level, health training received, age, and gender can all influence the quality of malaria diagnosis and treatment provided by CHVs in Western Kenya. However, other factors can influence the quality of malaria diagnosis and treatment provided by CHVs such as availability and quality of diagnostic tools and anti-malarial drugs, the support and supervision provided by healthcare professionals, and the context in which CHVs operate. To improve the quality of malaria diagnosis and treatment provided by CHVs, it is critical to consider all of these factors and design comprehensive interventions that address each community's unique challenges and opportunities, while also ensuring that CHVs are adequately trained, equipped, and supported to carry out their roles effectively.

## Supplementary Information

**Additional file 1: Table S1. **Categorization of Community health volunteers (CHVs) and febrile residents’ demographic information. **Table S2.** Association of CHVs demographics and quality of service.

## Data Availability

The dataset used in this study is available from the corresponding author upon request.

## Abbreviations

ACD: Active Case Detection
CI: Confidence Interval
DBS: Dried blood spots
CCMm: Community Case Mnagement of malaria
CHV: Community Health Volunteer
ICEMR: International Center of Excellence for Malaria Research
RDT: Rapid diagnosis test
RT-PCR: Real-time polymerase chain reaction
