# Supplementary material for: Community case management of malaria in Western Kenya: performance of community health volunteers in active malaria case surveillance
Source: Malar J. 2023 Mar 8;22:83. doi: 10.1186/s12936-023-04523-4 (PMC9993668; doi:10.1186/s12936-023-04523-4)
Supplement: Supplementary file 1 — Additional file 1: Table S1. Categorization of Community health volunteers (CHVs) and febrile residents’ demographic information. Table S2. Association of CHVs demographics and quality of service. [file 12936_2023_4523_MOESM1_ESM.docx]

**Additional file Table S1. Categorization of Community health volunteers (CHVs) and febrile residents’ demographic information**

| **Community health volunteer demographic information** | **Options** |
| --- | --- |
| 1. CHV’s age | 1. Less than 50 years; 2. 50 years and above |
| 2. Income generating activity | 1. Farmer; 2. Small scale business; 3. Others |
| 3. Years of experience | 1. Less than 10 years; 2. 10 years and above |
| 4. Level of education | 1. Primary education; 2. Secondary education |
| 5. Community health trainings | 1. Less than 10 trainings; 2. 10 trainings and above |
| **Febrile residents’ information** | **Options** |
| 6. Age | 1. <5 years old; 2. 5–15 years old; 3. >15 years old |
| 7. Fever days | 1. < 24 hours; 2. 24-72hours; 3. 72 hours |
| 8. Treatment-seeking methods | 1. Public hospital; 2. Private hospital; 3. Drug shops; 4. Traditional medication; 5. Do nothing |
| 9. Occupation | 1. Farmer; 2. Small scale business; 3. Unemployed; 4. Child younger than school age; 5. Others |
| 10. Travel history: having traveled outside the study zones within the previous two weeks | 1. Yes; 2. No |
| 11. Referral: referred to the health facilities for further treatment by the CHVs during the time of the visit | 1. Yes; 2. No |
| 12. Bednet usage: sleeping under a bednet the night before the survey | 1. Yes; 2. No |
| 13. Health insurance: having medical insurance coverage. | 1. Yes; 2. No |
| 14. Mode of transport to the health facility | 1. Walk; 2. Motorbike; 3. Vehicle |
| 15. Reasons for delay in seeking treatment | 1. Cost; 2. Distance; 3. Disease not severe; 4. Others |

**Additional file Table S2. Association of CHVs demographics and quality of service**

|  | **Details** | **CHVs age** | | **Sex** | | **Experience** | | **Education** | | **Training** | |
| --- | --- | --- | --- | --- | --- | --- | --- | --- | --- | --- | --- |
|  |  | < 50 years old | ≥ 50 years old | Male | Female | <10 years | ≥ 10 years | Primary | Secondary | <10 | ≥ 10 |
| **Correct use of Job aid** | Satisfactory (%) | 29 (100.0) | 40 (93.0) | 6 (85.7) | 63 (96.9) | 18 (94.7) | 1 (5.3) | 40 (97.6) | 29 (93.5) | 48 (100.0) | 21 (87.5) |
|  | Unsatisfactory (%) | 0 | 3 (7.0) | 1 (14.3) | 2 (3.1) | 51 (96.2) | 2 (3.8) | 1 (2.4) | 2 (6.5) | 0 | 3 (12.5) |
|  | Chi-square | 2.111 | | 1.988 | | 0.078 | | 0.712 | | 6.261 | |
|  | *p*-value | 0.146 | | 0.159 | | 0.780 | | 0.399 | | 0.01 | |
| **Classification of malaria symptoms** | Satisfactory (%) | 29 (100.0) | 43 (100.0) | 7 (100.0) | 65 (100) | 19 (100) | 53 (100) | 41 (100) | 31 (100) | 48 (100.0) | 24 (100.0) |
|  | Unsatisfactory (%) | 0 | 0 | 0 | 0 | 0 | 0 | 0 | 0 | 0 | 0 |
|  | Chi-square | - | | - | | - | | - | | - | |
|  | *p*-value | - | | - | | - | | - | | - | |
| **Experience with commodity stock out** | Satisfactory (%) | 28 (96.6) | 40 (93.0) | 6 (85.7) | 62 (95.4) | 18 (94.7) | 50 (94.3) | 39 (95.1) | 29 (93.5) | 47 (97.9) | 21(30.9) |
|  | Unsatisfactory (%) | 1(3.4) | 3 (7.0) | 1 (14.3) | 3 (4.6) | 1 (5.3) | 3 (5.7) | 2 (4.9) | 2 (6.5) | 1 (2.1) | 3 (12.5) |
|  | Chi-square | 0.411 | | 1.126 | | 0.004 | | 0.083 | | 3.309 | |
|  | *p*-value | 0.521 | | 0.289 | | 0.948 | | 0.773 | | 0.069 | |
| **Safety procedure** | Satisfactory (%) | 29 (100.0) | 41 (95.3) | 6 (85.7) | 64 (98.5) | 19 (100) | 51 (96.2) | 40 (97.6) | 30 (96.8) | 48 (100.0) | 22 (91.7) |
|  | Unsatisfactory (%) | 0 | 2 (4.7) | 1 (14.3) | 1 (1.5) | 0 | 2 (3.8) | 1 (2.4) | 1 (3.2) | 0 | 2 (8.3) |
|  | Chi-square | 1.387 | | 3.802 | | 0.737 | | 0.040 | | 4.114 | |
|  | *p*-value | 0.239 | | 0.051 | | 0.890 | | 0.841 | | 0.043 | |
